# Ti-24Nb-4Zr-8Sn Alloy Pedicle Screw Improves Internal Vertebral Fixation by Reducing Stress-Shielding Effects in a Porcine Model

**DOI:** 10.1155/2018/8639648

**Published:** 2018-02-08

**Authors:** Yang Qu, Shuang Zheng, Rongpeng Dong, Mingyang Kang, Haohan Zhou, Dezhi Zhao, Jianwu Zhao

**Affiliations:** Department of Orthopedics, The Second Hospital of Jilin University, Changchun 130041, China

## Abstract

To ensure the biomechanical properties of Ti-24Nb-4Zr-8Sn, stress-shielding effects were compared between Ti-24Nb-4Zr-8Sn and Ti-6Al-4V fixation by using a porcine model. Twelve thoracolumbar spines (T12–L5) of 12-month-old male pigs were randomly divided into two groups: Ti-24Nb-4Zr-8Sn (EG, *n* = 6) and Ti-6Al-4V (RG, *n* = 6) fixation. Pedicle screw was fixed at the outer edge of L4-5 vertebral holes. Fourteen measuring points were selected on the front of transverse process and middle and posterior of L4-5 vertebra. Electronic universal testing machine was used to measure the strain resistance of measuring points after forward and backward flexion loading of 150 N. Meanwhile, stress resistance was compared between both groups. The strain and stress resistance of measurement points 1, 2, 5, 6, 9, and 10–14 in Ti-24Nb-4Zr-8Sn fixation was lower than that of Ti-6Al-4V fixation after forward and backward flexion loading (*P* < 0.05). The strain and stress resistance of measurement points 3, 4, 7, and 8 was higher in Ti-24Nb-4Zr-8Sn fixation than that of Ti-6Al-4V fixation (*P* < 0.05). Stress-shielding effects of Ti-24Nb-4Zr-8Sn internal fixation were less than that of Ti-6Al-4V internal fixation. These results suggest that Ti-24Nb-4Zr-8Sn elastic fixation has more biomechanical goals than conventional Ti-6Al-4V internal fixation by reducing stress-shielding effects.

## 1. Introduction

With global population aging, the incidence of degenerative lumbar degeneration has increased [1]. Vertebral fusion has become a gold standard for the treatment of lumbar degenerative diseases in spine surgeons [2, 3]. Lumbar fusion of clinical application has been significantly improved in past decades, and rigid internal fixation technology has increased lumbar fusion rates [4, 5].

Low back pain induced by degenerative diseases is often experienced by the individuals beyond middle age. The standard treatment is decompression and interbody fusion, which is followed by a pedicle screw-rod system to stabilize spine [6, 7]. Conventional pedicle screw-rod system is made from the titanium alloy Ti-6A1-4V, which is biocompatible, corrosion resistant, bendable, and stiff enough to provide sufficient stability for spine [8]. However, the elastic modulus of Ti-6A1-4V (110 GPa) is much higher than that of cortical bone (18 GPa), and thus it changes spinal kinetics. After the implantation of Ti-6A1-4V, the motion of adjacent segments increases to compensate the reduced motion of implanted segments, which accelerate adjacent disc degeneration [9]. A stress-shielding effect, in which some stresses on vertebral bodies are shifted to pedicle-rod systems, decelerates intervertebral fusion and increases the looseness of pedicle screw. To eliminate these complications, some dynamic fixation systems have been developed. The most used one is the Dynesys Dynamic Stabilization System [10, 11], in which titanium alloy rods are replaced with polymolecular cords and spacers. However, the long-term clinical outcomes are not much better than expected [12].

Furthermore, due to strong stress-shielding effects caused by internal fixation, bone stress stimulation was significantly reduced, leading to bone reconstruction and absorption [13]. Biomechanical measurement of fusion techniques shows that all fusion will increase the stress of adjacent vertebrae [14]. Animal experiments also confirmed that strong internal fixation could cause stress occlusion and osteoporosis [15]. The increase of the stress near the segment after strong internal fixation accelerates the degeneration of adjacent segment disc [16, 17], which has become an urgent problem that needs to be solved after lumbar fusion.

In order to solve the problem caused by strong internal fixation, the researchers induce the concept of elastic fixation of spine and the premise of skeletal stability [18]. To reduce the load of the internal fixation and increase sharing load of spine, it is necessary to reduce the stress block and focus. At present, most researchers have applied intramedullary nailing or bone plate, joint replacement prosthesis implantation, and postoperative fixation of spine after internal fixation [19, 20]. However, the degree of occlusion remains unclear [21, 22]. The data show that there is no significant difference between the elastic internal and rigid internal fixation system in the therapy of lumbar degenerative lesions [23]. Low modulus beta-titanium alloys are hoped to provide good biocompatibility and alleviate stress-shielding effects. Titanium alloy Ti-6Al-4V is widely used for implants, which are characterized by high elastic modulus that may induce undesirable stress-shielding effects and toxicity [24]. Ti-24Nb-4Zr-8Sn alloy, as a low modulus (49 GPa), has been approved to a potential bone load-bearing implant material without toxicity [25].

However, the effects of Ti-24Nb-4Zr-8Sn and Ti-6Al-4V fixation on shielding stress were seldom compared. To ensure biomechanical properties of Ti-24Nb-4Zr-8Sn, this experiment is performed to compare the effects of Ti-24Nb-4Zr-8Sn and Ti-6Al-4V fixation on shielding stress. Before and after internal fixation, the strain and stress resistance values of the two kinds of internal fixation on vertebra was compared under the same flexion load.

## 2. Materials and Methods

### 2.1. Materials

**Figure 1:**
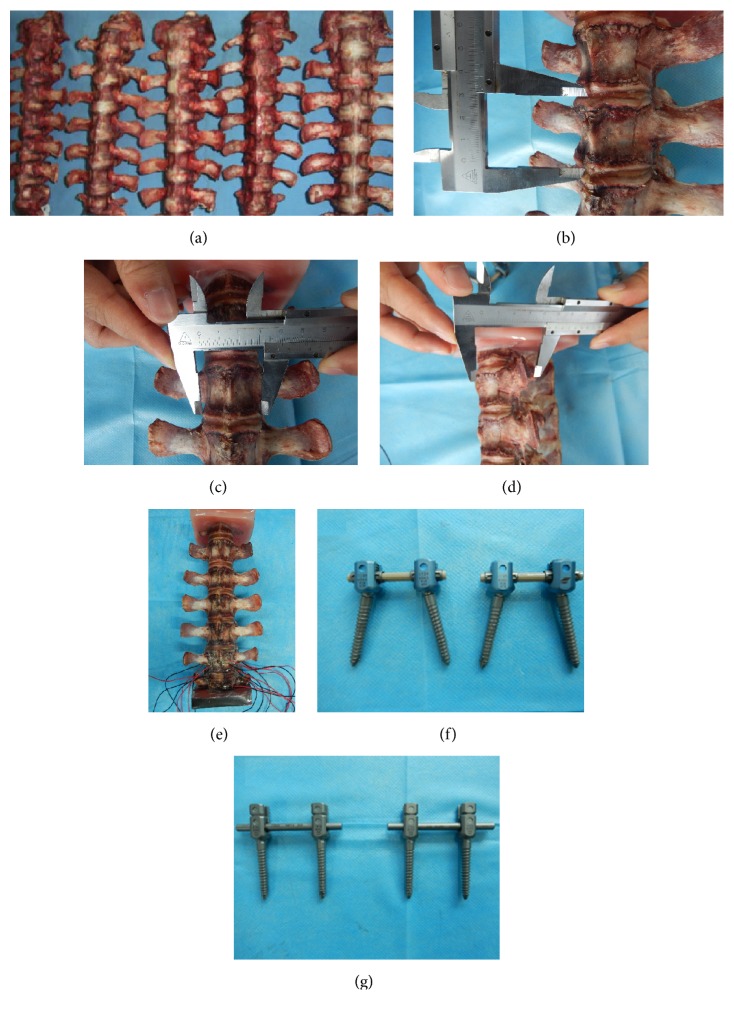
Specimen preparation. (a) Pig spine specimens with removal of muscle and other soft tissues. (b) Measurement of vertebral height. (c) Measurement of vertebral width. (d) Measurement of pedicle screw channel length. (e) Embedded specimen after fixation. (f) Ti-24Nb-4Zr-8Sn pedicle screw. (g) Ti-6A1-4V pedicle screw.

Twelve thoracolumbar spines (T12–L5) were obtained from 12-month male pigs. Before obtaining the specimens, all spines had no damage and deformity after being scanned by X-ray. All vertebra were randomly assigned into elastic Ti-24Nb-4Zr-8Sn (EG, *n* = 6) and rigid Ti-6Al-4V (RG, *n* = 6) fixation groups. Scalpel, posterior longitudinal ligament, anterior longitudinal ligament, and facet joint were removed by scalpel and spatula (Figure 1(a)). The specimens were numbered and basic dimensions were measured (Figures 1(b)–1(d)). Lumbar spine specimens were embedded and fixed with denture base resin solution.  Figure 1(e) showed the embedded species. Strain measurement was performed in both groups. The resistance of strain gauge was 121.4 ± 0.1 and gauge sensitivity coefficient was 2.14. Two kinds of pedicle screws Ti-24Nb-4Zr-8Sn and Ti-6Al-4V were used to fix vertebra as Figures 1(f) and 1(g) showed.

### 2.2. The Establishment of Measuring Points

With a finite element (FE) model and lumbar kinematic analysis according to earlier reports [26–28], biomechanical parameters with flexion loading were predicted. The geometry used in FE model of L4-5 was reconstructed across a porcine thoracolumbar spine. Before and after fixation at L4-5, the maximum strain and stress points were predicted after forward and backward flexion loading.

**Figure 2:**
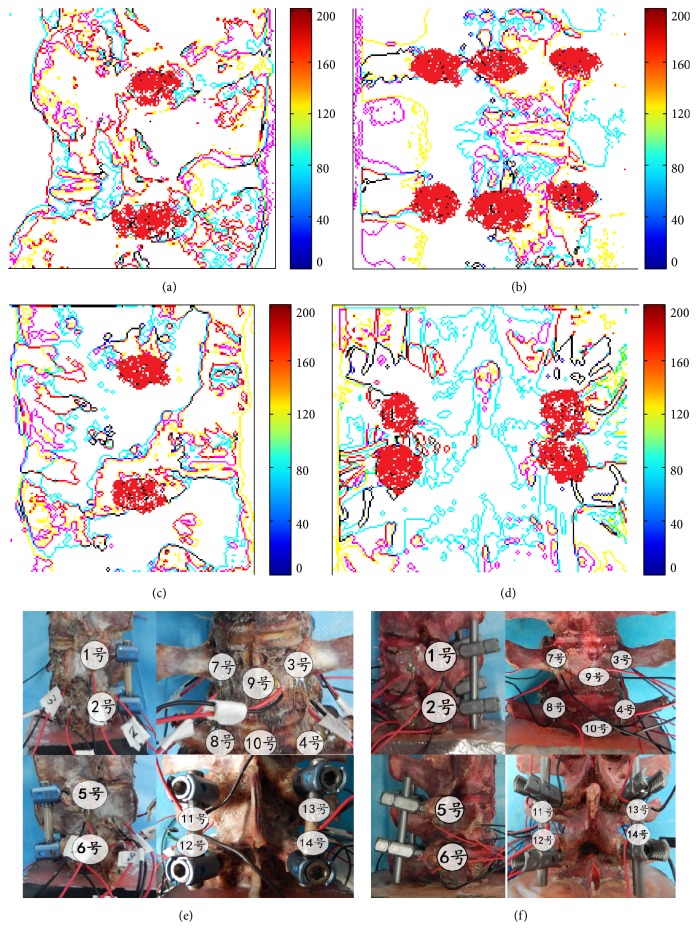
Measuring points predicted with finite element (FE) model and lumbar kinematic analysis. (a) Left L4-5 vertebra (measuring points numbers 1 and 2). (b) Middle L4-5 vertebra (measuring points numbers 3, 4, and 7–10). (c) Right L4-5 vertebra (measuring points 5 and 6). (d) Posterior L4-5 vertebra (measuring points 11–14). (e) Measuring points used in Ti-24Nb-4Zr-8Sn pedicle screw group. (f) Measuring points used in Ti-6A1-4V pedicle screw group. Number 1 point for left pedicle screw fixed at outside the hole of the lumbar vertebrae; number 2 point for left pedicle screw fixed at outside the hole of the lumbar vertebrae; number 3 points for pedicle screw fixed at left side of the transverse of waist L4 vertebral body. Number 5 point for pedicle screw fixed at outside the hole of vertebral vertebrae; number 6 points for right pedicle screw fixed below the ground of the lumbar vertebrae; number 7 points for right pedicle screw fixed at transverse sites of lumbar vertebrae; number 8 points for right pedicle screw fixed at transverse process; number 9 point for right pedicle screw fixed at front of waist L4 vertebral body; number 10 point for right pedicle screw fixed on the 10th vertebrae of the lumbar vertebrae. Number 11 point was at the junction of left fixation rod and L4 pedicle screw. Number 12 point was at the junction of left fixation rod and L5 pedicle screw. Number 13 point was at the junction of right fixation rod and L4 pedicle screw. Number 14 point was at the junction of right fixation rod and L5 pedicle screw.

A total of 14 measuring points with possible high strain and stress resistance were predicted with a finite element (FE) model and lumbar kinematic analysis. Measuring points numbers 1 and 2 were found at left faces of L4-5 vertebra (Figure 2(a)). Measuring points numbers 3, 4, and 7–10 were found at middle faces of L4-5 vertebra (Figure 2(b)). Measuring points 5 and 6 were found at right faces of L4-5 vertebra (Figure 2(c)). Measuring points 11–14 were found at the posterior of L4-5 vertebra (Figure 2(d)).  Figure 2(e) showed the measuring points used in Ti-24Nb-4Zr-8Sn group and Figure 2(f) showed measuring points used in Ti-6A1-4V group.

### 2.3. Measurement of Strain Resistance after Internal Fixation and Flexion Load

**Figure 3:**
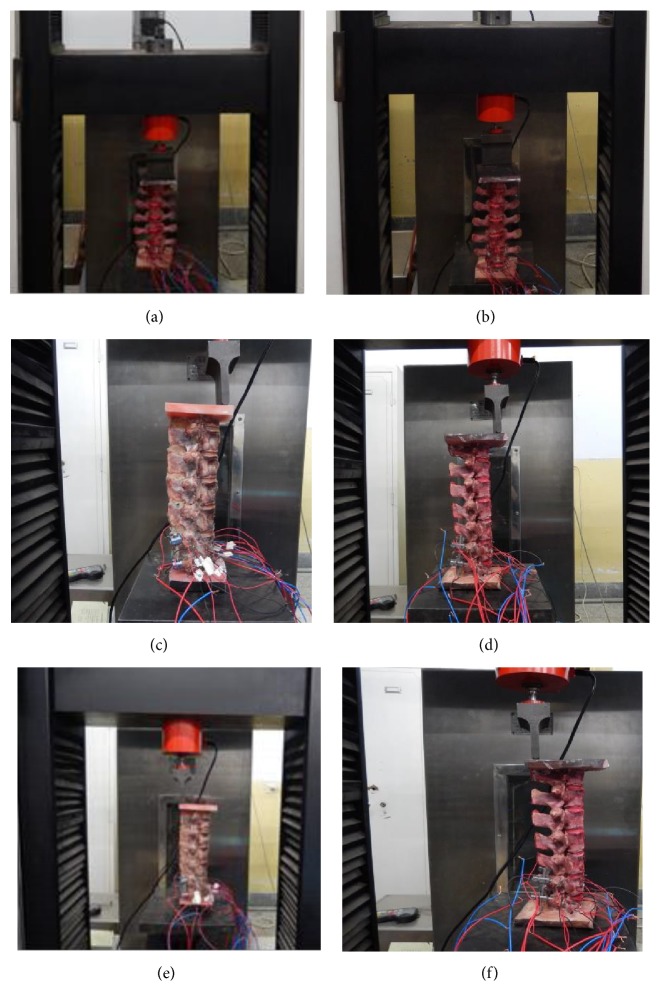
Strain resistance measurements. (a) After forward flexion load, strain resistance was measured before pedicle screw fixation. (b) After backward flexion load, strain resistance was measured before pedicle screw fixation. (c) After forward flexion load, strain resistance was measured in Ti-24Nb-4Zr-8Sn group after pedicle screw fixation. (d) After forward flexion load, strain resistance was measured in Ti-6A1-4V group after pedicle screw fixation. (e) After backward flexion load, strain resistance was measured in Ti-24Nb-4Zr-8Sn group after pedicle screw fixation. (f) After backward flexion load, strain resistance was measured in Ti-6A1-4V group after pedicle screw fixation.

The load was transmitted by the load cell with 300 N ranges, and the displacement was transmitted by photoelectric encoder. First of all, each specimen was installed in the test machine. Each specimen was loaded and unloaded 20 times for preconditioning processing. The strain gauge wire of each measuring point of the specimen was connected with the bridge arm of the dynamic resistance strain gauge junction box and bridged in a half way. The temperature compensation was adapted to the external condition. A load of 150 N was applied to the specimen at a load with increasing rate of 2 mm/min (the load applied in this experiment was within the physiological load range). The strain resistance of each measuring point was measured by dynamic resistance strain gauge. After forward and backward flexion load, strain resistance was measured before pedicle screw fixation (Figures 3(a) and 3(b)). After forward flexion load, strain resistance was measured in Ti-24Nb-4Zr-8Sn group (Figure 3(c)) and Ti-6A1-4V group (Figure 3(d)) after pedicle screw fixation. Strain resistance was also measured in. After backward flexion load, strain resistance was measured in Ti-24Nb-4Zr-8Sn group (Figure 3(e)) and Ti-6A1-4V group (Figure 3(f)) after pedicle screw fixation.

### 2.4. Each Group of Specimens of Pedicle Screw Fixation after Internal Fixation

As Figure 3 showed, pedicle screw was fixed before application of flexion load. Two kinds of pedicle screw were manufactured by medical instrument company (Chunlizhengda Co., Beijing, China) by using titanium alloy Ti-24Nb-4Zr-8Sn (49 GPa) or Ti-6A1-4V (110 GPa). The lumbar posterior (L4, L5) was fixed with Ti-24Nb-4Zr-8Sn fixator or Ti-6A1-4V fixator. Ten points for pedicle screw fixation were determined. In order to measure the internal rod fixation and the junction of the strain, four additional points were selected for the internal fixation and pedicle screw at the junction (11–14 points). The measurement points were shown in Figure 2. The measuring points were the same between the EG and the RG groups (Figures 2(e) and 2(f)).

For the measurement of the strain gauge after pedicle screw fixation: the same model, batch, sensitivity coefficient, and resistance of strain gauge were used. The stress measurement was performed to measure the strain of the rats under the flexion load before the internal fixation of pedicle screw under flexion load. Ti-24Nb-4Zr-8Sn and Ti-6A1-4V fixation were performed under 150-N flexion load shown in Figures 2(c)–2(f).

### 2.5. Stress Calculation

The stress formula is provided as follows:

(1) $$\begin{array}{c} \sigma =E\varepsilon , \end{array}$$

where *σ* is the stress, *ε* is the strain, and *E* is the elastic modulus. In this experiment, the modulus of rigid internal Ti-6A1-4V fixation system is 110 GPa, and the modulus of the Ti-24Nb-4Zr-8Sn elastic internal fixation system is about 70 GPa [29], and the elastic modulus of the lumbar vertebrae is 19.2 GPa [29].

### 2.6. Statistical Analysis Methods

SPSS software package 16.0 SPSS (Chicago, IL, USA) was used for data analysis. The difference between the two types of variable data of variance was analyzed by using Scheffe method and paired* t*-test. *P* < 0.05 for the statistical difference was significant.

## 3. Results

### 3.1. The Strain Resistance Values of Measuring Points between Ti-24Nb-4Zr-8Sn Internal Fixations Were Similar with Ti-6A1-4V before Internal Fixation

The strain resistance of Ti-24Nb-4Zr-8Sn fixation and Ti-6A1-4V fixation group was compared after flexion loading. Before alloy fixation, there was no significantly statistical difference for strain resistance after forward flexion loading (Table 1) or backward flexion loading (Table 2) between the EG and the RG groups (*P* > 0.05). The results suggested that the specimens were similar with the same strain resistance values of measuring points between the EG and the RG groups.

### 3.2. The Strain Resistance of Ti-24Nb-4Zr-8Sn Was Higher Than That of Ti-6A1-4V after Internal Fixation

Strain measurement showed that the strain resistance of the Ti-24Nb-4Zr-8Sn fixation group at 1, 2, 5, 6, 9, and 10–14 measuring was lower than that of the rigid internal fixation under forward flexion loading after fixation (Table 3, *P* < 0.05) and backward flexion loading (Table 4, *P* < 0.05). In contrast, strain resistance of Ti-24Nb-4Zr-8Sn elastic internal fixation at 3, 4, 7, and 8 measuring points was higher than that of Ti-6A1-4V fixation under forward flexion loading after fixation (Table 3, *P* < 0.05) and backward flexion loading (Table 4, *P* < 0.05).

### 3.3. Stress Resistance of Ti-24Nb-4Zr-8Sn Was Lower Than Ti-6A1-4V after Internal Fixation

The stress resistance of Ti-24Nb-4Zr-8Sn and Ti-6A1-4V internal fixation after flexion load was compared. The results showed that stress resistance of Ti-24Nb-4Zr-8Sn fixation was lower than that of Ti-6A1-4V fixation after forward flexion loading (Table 5, *P* < 0.05) and backward flexion loading (Table 6, *P* < 0.05) at measuring points 1, 2, 5, 6, 9, and 10–14. In contrast, stress resistance of Ti-24Nb-4Zr-8Sn fixation was higher than that of Ti-6A1-4V fixation after forward flexion loading (Table 5, *P* < 0.05) and backward flexion loading (Table 6, *P* < 0.05) at measuring points 3, 4, 7, and 8. All the results suggest that strain and stress resistance showed more stress-shielding effects in the RG group than in the EG group.

## 4. Discussion

The strain and stress resistance of the Ti-24Nb-4Zr-8Sn and Ti-6A1-4V fixation increased after flexion load, but the increase in Ti-6A1-4V group was greater than that of Ti-24Nb-4Zr-8Sn group. Strain resistance of 10 measuring points in Ti-6A1-4V group was higher than that of Ti-24Nb-4Zr-8Sn group (Tables 3 and 4, *P* < 0.05). Similarly, stress resistance of Ti-24Nb-4Zr-8Sn was also lower than that of Ti-6A1-4V group (Tables 5 and 6, *P* < 0.05). Normally, bone material with low modulus elasticity is less resistant to outside stress, while the material with high-modulus elasticity is more resistant to outside stress. The results showed that the stress-shielding effects of Ti-24Nb-4Zr-8Sn fixation were small.

Stress conduction can be caused by lumbar degeneration [16], whereas back pain and other symptoms can be reduced or even disappear if lumbar dynamic internal fixation system precisely limits the activities of the vertebral body [30]. Theoretically, lumbar dynamic stability system reduces adjacent segment degeneration and the stress on the intervertebral bone graft and promotes bone healing and spine recovery. However, high-level stress will cause spinal cord compression and induce nerve tissue damage [31]. Lumbar Ti-24Nb-4Zr-8Sn fixation system is a dynamic stability system made of titanium alloy and polyether ether ketone (PEEK) materials [32]. The materials are safe for patient health and have high strength, excellent mechanical properties, good self-lubricating, corrosion resistance, wear, and other characteristics [33]. Its ultralow elastic modulus in a simple bar-shaped design can achieve a satisfactory dynamic fixation effect. Ti-24Nb-4Zr-8Sn fixation system can reduce the stress resistance of titanium pedicle screw when the stress resistance acts on the elastic connecting rod with certain microaction because polyethylene fiber elastic connecting rod instead of rigid connecting rod with titanium alloys is used. The system reduces vertebral osteoporosis and pedicle screw loosening, fracture, and other risks and maintains intervertebral space and active function.

The load of the segment slows down the degeneration of adjacent segment discs. After the internal fixation of the spine, biomechanical properties of the column are restored, the load sharing of the internal fixation system is reduced, and the reduction of internal fixation load is important to avoid fixation failure [34]. Selective stress occlusion is another way to address stress-induced osteoporosis [35]. Vertebral compression stress stimulates bone growth and strengthens. Selective stress occlusion makes the internal fixation mainly against lateral bending, rotation and shear stress, reducing the compressive stress on internal fixation, thereby increasing the compressive stress of the vertebral body and compressive stress of vertebra. All these results can improve mechanical performance. Ti-24Nb-4Zr-8Sn fixation improved patient's postoperative symptoms significantly when compared with fusion surgery with Ti-6A1-4V [36].

Lumbar Ti-24Nb-4Zr-8Sn internal fixation system has the following advantages: retaining motion function of the surgical segment while reducing the stress resistance of adjacent segments, delaying the occurrence of degeneration and even less damage to the disc, providing sufficient stability while retaining a certain degree of activity of lumbar spine, and delaying the occurrence of adjacent segment degeneration. This experiment is done by establishing a biomechanical model with Ti-24Nb-4Zr-8Sn fixation. The results showed that the effects of stress occlusion caused by Ti-24Nb-4Zr-8Sn fixation were less than those caused by Ti-6A1-4V fixation.

*Limitations of the Present Work*. There were some limitations of the present study: Ti-24Nb-4Zr-8Sn fixation lacks long-term randomized controlled outcome, and long-term clinical stability, screws loosening, fatigue of screw, and connecting rods remain unclear; the specimens used for the lumbar spine may cause different results when compared with other specimens; only Ti-24Nb-4Zr-8Sn and Ti-6A1-4V fixation were used to compare stress-shielding effects. In the posterior extension, compression, left and right bending, and stress need to be further analyzed in the future.

## 5. Conclusion

After fixation, strain and stress resistance of measurement points 1, 2, 5, 6, 9, and 10–14 in Ti-24Nb-4Zr-8Sn fixation was lower than that of Ti-6Al-4V fixation after forward and backward flexion loading. In contrast, the strain and stress resistance of measurement points 3, 4, 7, and 8 was higher in Ti-24Nb-4Zr-8Sn fixation than that of Ti-6Al-4V fixation. Stress-shielding effects of Ti-24Nb-4Zr-8Sn fixation were less than those of Ti-6Al-4V fixation. Ti-24Nb-4Zr-8Sn fixation has more biomechanical properties than conventional Ti-6Al-4V fixation by reducing stress-shielding effects.

## Figures and Tables

**Table 1:** The strain resistance of Ti-24Nb-4Zr-8Sn and Ti-6A1-4V fixation groups under forward flexion loading before fixation (*με*, ×10^−6^).

Measuring points	Sample 1	Sample 2	Sample 3	Sample 4	Sample 5	Sample 6	Mean ± SD
1							
EG	130	131	133	127	124	129	129 ± 3
RG	138	145	133	140	137	143	139 ± 4
2							
EG	126	125	129	130	134	127	129 ± 3
RG	127	134	139	136	129	135	133 ± 5
3							
EG	155	148	159	161	155	150	155 ± 5
RG	157	153	156	161	159	164	158 ± 4
4							
EG	165	156	160	163	152	157	159 ± 5
RG	165	159	163	157	160	159	161 ± 3
5							
EG	128	123	127	131	130	129	128 ± 3
RG	126	135	133	130	128	129	130 ± 3
6							
EG	125	127	129	132	128	123	127 ± 3
RG	129	126	134	133	125	127	129 ± 4
7							
EG	161	168	164	158	167	159	163 ± 4
RG	159	164	162	159	169	168	164 ± 4
8							
EG	172	169	159	177	175	171	171 ± 6
RG	170	165	170	157	165	172	167 ± 5
9							
EG	188	195	193	189	191	192	191 ± 3
RG	184	188	197	189	196	190	191 ± 5
10							
EG	191	186	187	192	196	191	191 ± 4
RG	187	190	186	195	188	193	190 ± 4
11							
EG	192	189	202	193	188	197	194 ± 5
RG	189	191	200	195	192	201	195 ± 5
12							
EG	200	197	195	199	192	189	195 ± 4
RG	196	188	190	200	196	196	194 ± 4
13							
EG	188	194	192	197	201	196	195 ± 4
RG	192	198	188	192	205	200	196 ± 6
14							
EG	194	199	189	193	194	187	193 ± 4
RG	189	195	192	183	187	195	190 ± 5

**Table 2:** The strain resistance of Ti-24Nb-4Zr-8Sn and Ti-6A1-4V fixation groups under backward flexion loading before fixation (*με*, ×10^−6^).

Measuring points	Sample 1	Sample 2	Sample 3	Sample 4	Sample 5	Sample 6	Mean ± SD
1							
EG	−200	−189	−201	−205	−192	−197	−197 ± 6
RG	−203	−187	−197	−199	−201	−197	−197 ± 6
2							
EG	−205	−196	−198	−192	−193	−201	−198 ± 5
RG	−204	−196	−193	−201	−195	−203	−199 ± 5
3							
EG	−167	−174	−168	−172	−173	−169	−171 ± 3
RG	−177	−176	−168	−175	−172	−169	−173 ± 4
4							
EG	−178	−169	−177	−169	−174	−170	−173 ± 4
RG	−173	−169	−175	−174	−178	−165	−172 ± 5
5							
EG	−198	−202	−199	−195	−201	−197	−199 ± 3
RG	−197	−194	−192	−196	−202	−200	−197 ± 4
6							
EG	−207	−209	−196	−203	−208	−195	−203 ± 6
RG	−204	−202	−197	−193	−196	−199	−199 ± 4
7							
EG	−150	−162	−155	−157	−159	−161	−157 ± 4
RG	−155	−161	−157	−154	−159	−163	−158 ± 3
8							
EG	−167	−178	−168	−172	−170	−169	−171 ± 4
RG	−173	−175	−166	−161	−167	−169	−169 ± 5
9							
EG	−128	−136	−130	−132	−129	−135	−132 ± 3
RG	−124	−133	−131	−127	−130	−128	−129 ± 3
10							
EG	−134	−137	−133	−137	−135	−130	−134 ± 3
RG	−132	−130	−137	−135	−129	−131	−132 ± 3
11							
EG	−219	−216	−233	−229	−230	−227	−226 ± 7
RG	−214	−213	−225	−231	−219	−232	−222 ± 8
12							
EG	−231	−227	−218	−231	−237	−226	−228 ± 6
RG	−228	−231	−221	−242	−234	−220	−229 ± 8
13							
EG	−217	−241	−229	−236	−224	−238	−231 ± 9
RG	−220	−233	−231	−232	−231	−229	−229 ± 5
14							
EG	−224	−223	−236	−240	−229	−237	−232 ± 7
RG	−233	−220	−231	−236	−238	−241	−233 ± 7

**Table 3:** The strain resistance of -24Nb-4Zr-8Sn and Ti-6A1-4V fixation groups under forward flexion loading after fixation (*με*, ×10^−6^).

Measuring points	Sample 1	Sample 2	Sample 3	Sample 4	Sample 5	Sample 6	Mean ± SD
1							
EG	149	152	147	152	155	161	152.67 ± 4.93^*∗*^
RG	188	179	187	193	196	189	188.66 ± 5.82
2							
EG	155	160	153	145	162	154	154.83 ± 5.98^*∗*^
RG	193	196	201	186	193	182	191.83 ± 6.85
3							
EG	134	131	129	133	126	130	130.50 ± 2.88^*∗*^
RG	105	97	99	102	106	98	101.17 ± 3.76
4							
EG	137	126	124	135	129	135	131.00 ± 5.40^*∗*^
RG	107	103	104	99	103	101	102.83 ± 2.71
5							
EG	156	147	149	153	160	156	153.50 ± 4.85^*∗*^
RG	203	192	183	179	197	185	189.83 ± 9.13
6							
EG	152	154	148	157	155	163	154.83 ± 5.04^*∗*^
RG	189	198	195	184	204	197	194.50 ± 7.06
7							
EG	132	125	129	136	127	133	130.33 ± 4.08^*∗*^
RG	96	103	105	102	98	96	100.00 ± 3.85
8							
EG	134	129	124	131	135	137	131.67 ± 4.72^*∗*^
RG	99	97	108	107	106	103	103.33 ± 4.50
9							
EG	129	135	127	132	131	135	131.50 ± 3.21^*∗*^
RG	159	165	161	157	163	162	161.17 ± 2.86
10							
EG	132	130	128	135	134	129	131.33 ± 2.80^*∗*^
RG	164	157	163	160	159	166	161.50 ± 3.39
11							
EG	119	122	125	117	127	133	123.83 ± 5.81^*∗*^
RG	152	149	162	153	148	157	153.50 ± 5.24
12							
EG	126	122	132	124	131	118	125.50 ± 5.36^*∗*^
RG	160	157	155	159	152	149	155.33 ± 4.23
13							
EG	125	124	127	123	118	124	123.50 ± 302^*∗*^
RG	148	154	152	157	161	156	154.67 ± 4.48
14							
EG	129	116	119	129	125	118	122.67 ± 5.75^*∗*^
RG	154	159	149	153	154	147	152.67 ± 4.23

*Note*. ^*∗*^*P* < 0.05  versus an RG group. Strain values are positive (tensile) due to elongation caused by forward flexion load.

**Table 4:** The strain resistance of Ti-24Nb-4Zr-8Sn and Ti-6A1-4V fixation groups under backward flexion loading after fixation (*με*, ×10^−6^).

Measuring points	Sample 1	Sample 2	Sample 3	Sample 4	Sample 5	Sample 6	Mean ± SD
1							
EG	−245	−254	−249	−257	−261	−258	−254 ± 6^*∗*^
RG	−310	−305	−319	−304	−318	−301	−310 ± 8
2							
EG	−251	−260	−257	−249	−255	−253	−254 ± 4^*∗*^
RG	−321	−317	−306	−320	−309	−305	−313 ± 7
3							
EG	−109	−127	−130	−121	−129	−123	−123 ± 8^*∗*^
RG	−92	−96	−89	−90	−88	−91	−91 ± 3
4							
EG	−121	−123	−107	−114	−125	−110	−117 ± 7^*∗*^
RG	−87	−98	−85	−92	−96	−93	−92 ± 5
5							
EG	−260	−254	−257	−249	−262	−255	−256 ±5^*∗*^
RG	−314	−321	−317	−305	−321	−318	−316 ± 6
6							
EG	−257	−248	−263	−246	−250	−259	−254 ± 7^*∗*^
RG	−309	−307	−312	−318	−317	−311	−312 ± 4
7							
EG	−124	−114	−131	−109	−123	−122	−121 ± 8^*∗*^
RG	−87	−84	−95	−97	−88	−93	−91 ± 5
8							
EG	−127	−109	−128	−117	−125	−120	−121 ± 7^*∗*^
RG	−94	−89	−90	−93	−97	−85	−91 ± 4
9							
EG	−87	−83	−91	−82	−88	−90	−87 ± 4^*∗*^
RG	−98	−117	−104	−117	−103	−119	−110 ± 9
10							
EG	−82	−90	−87	−84	−81	−89	−86 ± 4^*∗*^
RG	−105	−110	−109	−121	−105	−114	−111 ± 6
11							
EG	−145	−151	−155	−150	−163	−158	−154 ± 6^*∗*^
RG	−199	−196	−213	−209	−210	−207	−206 ± 7
12							
EG	−156	−148	−161	−157	−153	−148	−154 ± 5^*∗*^
RG	−211	−207	−198	−211	−217	−206	−208 ± 6
13							
EG	−153	−147	−162	−147	−165	−154	−155 ± 8^*∗*^
RG	−197	−221	−209	−216	−204	−218	−211 ± 9
14							
EG	−160	−155	−153	−156	−161	−146	−155 ± 5^*∗*^
RG	−204	−203	−216	−220	−209	−217	−212 ± 7

*Note*. ^*∗*^*P* < 0.05  versus an RG group. Strain values are negative due to compressive pressure caused by backward flexion load.

**Table 5:** The stress resistance of Ti-24Nb-4Zr-8Sn and Ti-6A1-4V fixation groups under forward flexion loading after fixation (MPa).

Measuring points	Sample 1	Sample 2	Sample 3	Sample 4	Sample 5	Sample 6	Mean ± S.D
1							
EG	2.86	2.92	2.82	2.92	2.98	3.09	2.93 ± 0.09^*∗*^
RG	3.61	3.44	3.59	3.71	3.76	3.63	3.62 ± 0.11
2							
EG	2.98	3.07	2.94	2.78	3.11	2.96	2.97 ± 0.12^*∗*^
RG	3.71	3.76	3.86	3.57	3.71	3.49	3.68 ± 0.13
3							
EG	2.57	2.52	2.48	2.55	2.42	2.5	2.51 ± 0.05^*∗*^
RG	2.02	1.86	1.9	1.96	2.04	1.88	1.94 ± 0.08
4							
EG	2.63	2.42	2.38	2.59	2.48	2.59	2.52 ± 0.10^*∗*^
RG	2.05	1.98	2	1.9	1.98	1.94	1.98 ± 0.05
5							
EG	3	2.82	2.86	2.94	3.07	3	2.95 ± 0.09^*∗*^
RG	3.9	3.69	3.51	3.44	3.78	3.55	3.65 ± 0.18
6							
EG	2.92	2.96	2.84	3.01	2.98	3.13	2.97 ± 0.10^*∗*^
RG	3.63	3.8	3.74	3.53	3.92	3.78	3.73 ± 0.14
7							
EG	2.53	2.4	2.48	2.61	2.44	2.55	2.50 ± 0.08^*∗*^
RG	1.84	1.98	2.02	1.96	1.88	1.84	1.92 ± 0.08
8							
EG	2.57	2.48	2.38	2.52	2.59	2.63	2.53 ± 0.09^*∗*^
RG	1.9	1.86	2.07	2.05	2.04	1.98	1.98 ± 0.09
9							
EG	2.48	2.59	2.44	2.53	2.52	2.59	2.53 ± 0.06^*∗*^
RG	3.05	3.17	3.09	3.01	3.13	3.11	3.09 ± 0.05
10							
EG	2.53	2.5	2.46	2.59	2.57	2.48	2.52 ± 0.05^*∗*^
RG	3.15	3.01	3.13	3.07	3.05	3.19	3.01 ± 0.06
11							
EG	8.97	8.79	9.56	9.03	8.73	9.26	9.06 ± 0.31^*∗*^
RG	13.09	13.42	13.75	12.87	13.97	14.63	13.62 ± 0.64
12							
EG	9.44	9.26	9.15	9.38	8.97	8.79	9.17 ± 0.25^*∗*^
RG	13.86	13.42	14.52	13.64	14.41	12.98	13.80 ± 0.59
13							
EG	8.73	9.09	8.97	9.26	9.5	9.2	9.13 ± 0.26^*∗*^
RG	13.75	13.64	13.97	13.53	12.98	13.64	13.58 ± 0.33
14							
EG	9.09	9.38	8.79	9.03	9.09	8.67	9.01 ± 0.25^*∗*^
RG	14.19	12.76	13.09	14.19	13.75	12.98	13.49 ± 0.63

*Note*. ^*∗*^*P* < 0.05  versus an RG group. Stress values are positive (tensile) due to elongation caused by forward flexion load.

**Table 6:** The stress resistance of Ti-24Nb-4Zr-8Sn and Ti-6A1-4V fixation groups under backward flexion loading after fixation (MPa).

Measuring points	Sample 1	Sample 2	Sample 3	Sample 4	Sample 5	Sample 6	Mean ± SD
1							
EG	−4.7	−4.88	−4.78	−4.93	−5.01	−4.95	−4.88 ± 0.15^*∗*^
RG	−5.95	−5.86	−6.12	−5.84	−6.11	−5.78	−5.94 ± 0.13
2							
EG	−4.82	−4.99	−4.93	−4.78	−4.9	−4.86	−4.88 ± 0.07^*∗*^
RG	−6.16	−6.09	−5.88	−6.14	−5.93	−5.86	−6.01 ± 0.14
3							
EG	−2.09	−2.44	−2.5	−2.32	−2.48	−2.36	−2.37 ± 0.15^*∗*^
RG	−1.77	−1.84	−1.71	−1.73	−1.69	−1.75	−1.75 ± 0.05
4							
EG	−2.32	−2.36	−2.05	−2.19	−2.4	−2.11	−2.24 ± 0.14^*∗*^
RG	−1.67	−1.88	−1.63	−1.77	−1.84	−1.79	−1.76 ± 0.10
5							
EG	−4.99	−4.88	−4.93	−4.78	−5.03	−4.9	−4.92 ± 0.09^*∗*^
RG	−6.03	−6.16	−6.09	−5.86	−6.16	−6.11	−6.07 ± 0.11
6							
EG	−4.93	−4.76	−5.05	−4.72	−4.8	−4.97	−4.87 ± 0.13^*∗*^
RG	−5.93	−5.89	−5.99	−6.11	−6.09	−5.97	−6.00 ± 0.09
7							
EG	−2.38	−2.19	−2.52	−2.09	−2.36	−2.34	−2.31 ± 0.15^*∗*^
RG	−1.67	−1.61	−1.82	−1.86	−1.69	−1.79	−1.74 ± 0.10
8							
EG	−2.44	−2.09	−2.46	−2.25	−2.4	−2.3	−2.32 ± 0.14^*∗*^
RG	−1.8	−1.71	−1.73	−1.79	−1.86	−1.63	−1.75 ± 0.08
9							
EG	−1.67	−1.59	−1.75	−1.57	−1.69	−1.73	−1.67 ± 0.07^*∗*^
RG	−1.88	−2.25	−2	−2.25	−1.98	−2.28	−2.11 ± 0.17
10							
EG	−1.57	−1.73	−1.67	−1.61	−1.56	−1.71	−1.64 ± 0.07^*∗*^
RG	−2.02	−2.11	−2.09	−2.32	−2.02	−2.19	−2.13 ± 0.11
11							
EG	−11.74	−11.56	−12.57	−12.33	−12.39	−12.21	−12.13 ± 0.40^*∗*^
RG	−15.95	−16.61	−17.05	−16.5	−17.93	−17.38	−16.90 ± 0.70
12							
EG	−12.45	−12.21	−11.68	−12.45	−12.8	−12.15	−12.29 ± 0.38^*∗*^
RG	−17.16	−16.28	−17.71	−17.27	−16.83	−16.28	−16.92 ± 0.57
13							
EG	−11.62	−13.04	−12.33	−12.74	−12.04	−12.86	−12.44 ± 0.54^*∗*^
RG	−16.83	−16.17	−17.82	−16.17	−18.15	−16.94	−17.01 ± 0.83
14							
EG	−12.04	−11.98	−12.74	−12.98	−12.33	−12.8	−12.48 ± 0.42^*∗*^
RG	−17.6	−17.05	−16.83	−17.16	−17.71	−16.06	−17.07 ± 0.60

*Note*. ^*∗*^*P* < 0.05  versus an RG group. Stress values are negative due to compressive pressure caused by backward flexion load.
